# Impact of leaf trichomes of tomatoes and weeds on the host selection and developmental bioassays of *Bemisia tabaci* Q and A cryptic species

**DOI:** 10.1016/j.heliyon.2023.e20077

**Published:** 2023-09-12

**Authors:** Md Mostafizur Rahman Shah, Zhongkai Zhang, Jian Hu, Ahmed Gaber, Akbar Hossain

**Affiliations:** a Ministry of Agriculture Key Lab of Southwestern Crop Gene Resources and Germplasm Innovation, Yunnan Provincial Key Lab of Agricultural Biotechnology, Biotechnology and Germplasm Resources Institute, Yunnan Academy of Agricultural Sciences, 650223, Kunming, China; b Division of Entomology, Bangladesh Wheat and Maize Research Institute, Dinajpur, 5200, Bangladesh; c Department of Biology, College of Science, Taif University, P.O. Box 11099, Taif, 21944, Saudi Arabia; d Division of Soil Science, Bangladesh Wheat and Maize Research Institute, Dinajpur, 5200, Bangladesh

**Keywords:** *B*. *tabaci* Q, *B. tabaci* A, Host selection, Tomato, Weed, Leaf trichomes

## Abstract

The whiteflies of *Bemisia tabaci* complex, composed of >44 cryptic species, are economically important pests of tomatoes for their direct feeding and virus transmission. The present study aimed to evaluate the impact of leaf trichomes on the host selection and development of whitefly; comparative invasiveness between *B. tabaci* Q and A cryptic species; and the ability of weeds as hosts of the population of insect whitefly. We carried out our investigation through adult host selection and oviposition in multi-choice conditions, immature development and survival, and adult survival and oviposition in no-choice conditions. We investigated leaf trichomes type and densities on the leaves of four tomato varieties and two weed species. Results showed that the leaf trichomes of tomatoes and weeds impact the host selection and immature development differently on the cryptic species *B. tabaci* Q and A. In the multi-choice case, *B. tabaci* Q adults preferred tomato varieties Ao-Ni-Er and He-Fen for both settling and oviposition whereas *B. tabaci* A preferred Ao-Ni-Er, He-Fen, and Billy-Goat-Weed for settling but oviposited more eggs on both weed species Billy-Goat-Weed and False-Mallow. Both *B. tabaci* Q and A adults refused Ye-Sheng either settling or oviposition. In the case of immature development, *B. tabaci* Q developed faster than *B. tabaci* A. Concerning plant, *B. tabaci* Q developed faster on Ao-Ni-Er, He-Fen and Billy-Goat-Weed but *B. tabaci* A on Billy-Goat-Weed, False-Mallow and Ao-Ni-Er. The immature survival of Q was higher than that of A. Immature of *B. tabaci* Q survived well (68.6–86.8%) on all plants except Ye-Sheng (49.3%) but *B. tabaci* A survived very less (0–17.6%) on any tomatoes where 70.4% on Billy-Goat-Weed and 60.5% on False-Mallow. After seven days of adult infestation, both *B. tabaci* Q and A died on Ye-Sheng where 52.5–78.1% survivorships were observed on other plants. In seven days, *B. tabaci* Q laid more eggs compared to *B. tabaci* A. Considering the plants, both species laid more eggs on Ao-Ni-Er, He-Fen and False-Mallow, whereas the lowest number was laid on Ye-Sheng. The highest number of glandular trichome Type IV was observed on Ye-Sheng which showed resistance against both *B. tabaci* Q and A cryptic species. The cryptic species *B. tabaci* Q showed a wider range adaptation ability on plants than that of A. Weeds can play a significant role as an infestation source of whiteflies to tomatoes and other crops. These findings suggest that glandular trichomes may be used in plant breeding programmes for the development of whitefly-resistant crop cultivars.

## 1 Introduction

The whitefly, *Bemisia tabaci* Gennadius (Hemiptera: Aleyrodidae), is one of the notorious pest insects of vegetables, broadleaf field crops, ornamentals and some fruits in both open and protected crop fields worldwide [[1], [2], [3]]. It infests more than 600 plant species and can spread frequently into new territories [[4], [5], [6]]. In 1949, *B. tabaci* was reported for the first time in China [7] though it was considered a pest from the mid-1990s [8,9] and in the late 1990s it became a devastating pest [[10], [11], [12]]. This insect can damage plants in direct and indirect ways. Direct feeding of phloem sap by both adult and immature causes stunted plant growth and reduces yield. During sap-sucking, sometimes, injecting phytotoxins inside plants cause physiological disorders [2]. Indirectly, the transmission of the virus causes viral diseases and through honeydew secretion causes black sooty mould, impeding the functioning of plant photosynthesis [2,13]. These damages increase with time being and cause economic losses significantly. Reports show that millions of dollars have been lost due to feeding damages and viral diseases by *B. tabaci* [14,15].

*B. tabaci* is currently considered a species complex of races [11,16,17] showing different behaviour regarding their host preference, fecundity, environmental adaptation, and efficiency of virus transmission [11,[18], [19], [20]]. Many biotypes of *B. tabaci* have been identified in different regions of the world [17,21] of which Q and A are the considerable ones. These complex insects are multivoltine in nature and have no diapause or quiescent stage. Consequently, populations are sustained through the continual exploitation of multiple host resources over the annual cycle.

Tomato, *Solanum lycopersicum* L. is considered to be one of the economically important crops in most countries over the world. It is an advantageous crop due to its high yield within a short duration and its significant nutritional value. But many obstacles have been observed during tomato cultivation where insect pests and diseases are major ones. *B. tabaci* is one of the devastating pests of tomato plants as a direct feeder and as the vector of many viral diseases. Tomato Yellow Leaf Curl Virus (TYLCV) was first reported in Israel and caused by *B. tabaci* in 1939–40 [22]. Since then TYLCV was spreading to every region in the world and becoming a major limiting factor in tomato production [22]. The whiteflies of the Aleyrodidae family transmit 114 species of plant viruses while only *B. tabaci* transmits 111 of them [2]. Among the whitefly-transmit viruses, 90% are in the genus of *Begomovirus*, 6% are in the genus of *Crinivirus*, and the rest 4% are in the genera of *Carlavirus*, *Closterovirus* or *Ipomovirus* [2].

Trichomes are small protrusions of epidermal origin as extrusions or appendages on the leaf surfaces or other plant parts [23]. It functions as stress resistance, especially insect pest defense. Trichomes are usually hair-like structured shapes but sometimes appear as scales, buds or papillae and range from short to long with unicellular to multicellular structures [24]. Trichomes on tomato leaves or plants are mostly hair-like in shape with eight different types including four glandular types [25]. Glandular trichomes typically consist of one or more glandular cells that can produce, store and exude some specialized metabolites [26]. These metabolites, especially acyl-sugars, indoles, methyl ketones, phenolic compounds, and terpenoids have defensive properties against herbivores [25]. Tomato glandular trichomes can generate specialized metabolites that have defensive mechanisms against many insect pests and have properties in attracting natural enemies of herbivores known as beneficial insects [27,28].

Both direct and indirect interactions occur between weeds and many herbivores. Direct interaction occurs when insects directly feed on weeds whereas indirect interaction occurs through altering the ecosystem of the crop field by the weeds or acting as a host of predatory and or parasitic insects [29]. Many weeds play a role as the host plant for feeding insects like whiteflies. Accordingly, weeds that are intimately associated with the crops are most important in hosting insects that are more invasive than crops [30]. Adjacent places of crop fields more likely irrigation ditches, crop field borders, and fences are common areas for weed growth that can perform as the source of insect inoculum [31]. Nonetheless, weeds play a vital role in the overwintering of insects during the cropping off-season. Research reports showed that weeds are the alternate hosts and reservoirs of many Gemini viruses that transmit through insects and cause diseases in many crops [[32], [33], [34], [35]]. Billy-Goat-Weed (*Ageratum conyzoides*), a common weed in the agricultural field is often shows striking yellow vein symptoms and known to be the natural host of twelve *Begomovirus* species [36]. False-Mallow (*Malvastrum coromandelianum*) is an alternative host of Tomato yellow leaf curl China virus (TYLCCNV) and contributes to the epidemiology of tomato yellow leaf curl diseases [37]. *M. coromandelianum* also harbours Malvastrum leaf curl Guangdong virus (MLCuGdV) which was infected by whitefly transmission [38].

Although a large number of studies have been conducted on the bioassays of *Bemisia tabaci* Q on various host plants [[39], [40], [41], [42], [43], [44], [45], [46]], but no or few studies have been carried out on tomato varieties and weed species, and even the bioassays of *B. tabaci* A cryptic species. The present study aimed to assess comparative invasiveness between *B. tabaci* Q and A cryptic species; the impact of leaf trichomes on the host selection, oviposition, survival and immature development of *B. tabaci* Q and A cryptic species; and the role of weeds as hosts to support overwintering whiteflies.

## 2 Materials and methods

### 2.1 Host plants and whitefly culture

**Fig. 1:**
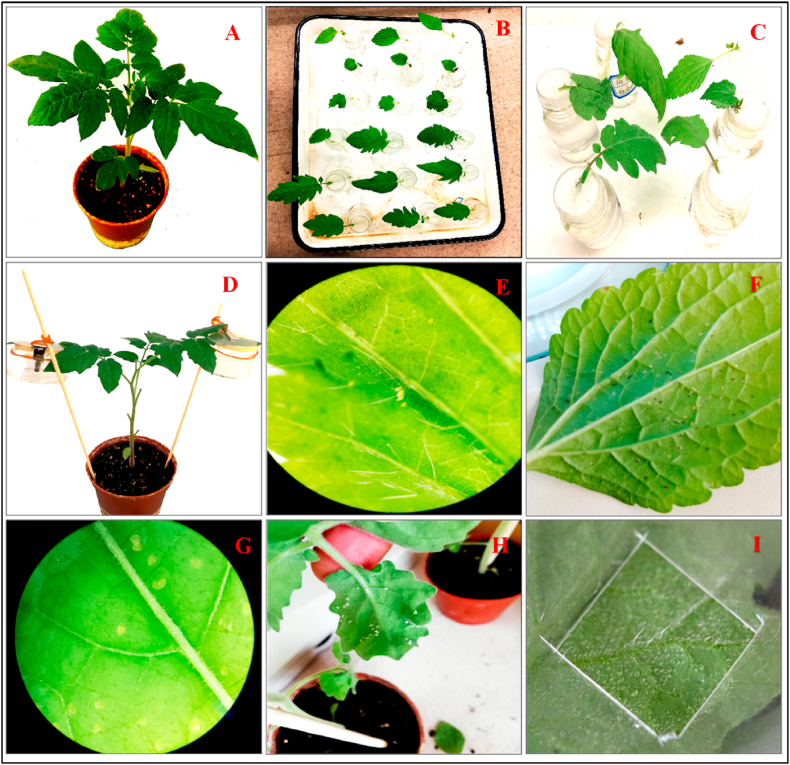
**(A):** Plant ready to use; **(B):** Preparing leaves for assays; **(C):** Adult host selection in multi-choice condition; **(D):** Adult whiteflies confined in clip-cage onto the abaxial leaf surface; **(E):** Whitefly eggs; **(F):** Nymph development on leaf surface; **(G):** Microscopic image of nymph development; **(H):** Whitefly adults died on Ye-Sheng leaf and **(I):** Leaf trichome identification and counting.

Four varieties, Ao-Ni-Er (ANE), He-Fen (HF), He-Zuo (HZ), Ye-Sheng (YS) of tomato, *S. lycopersicum* L. (Solanaceae) and two weed species, Billy-Goat-Weed, *Ageratum conyzoides* L. (Asteraceae), and False-Mallow, *Malvastrum coromandelianum* L. (Garcke), (Malvaceae) were used as host plants in this study. Host plant seeds were germinated and seedlings were raised in the plastic pot on a mixer of peat moss, vermiculite, and perlite at 5:1:1 ratio v/v inside the controlled room at the temperature of 26 ± 1 °C, with RH of 60 ± 5%, and photoperiod of 16 L:8D h with the light intensity of 1400–1725 lux [47]. The plant seedlings were individually transplanted in 10 cm diameter plastic pots with the same potting mix for experimental observations. Bioassays were carried out in an insectarium with prevailing natural temperature (day: 22–26 °C, night: 18–20 °C) and humidity. In this study for all bioassays, plants were used at the five to six true leaves stage, and the second and third upper leaves of the plant (fully expanded leaves) were used (Fig. 1(A)).

Whiteflies, the cryptic species Q and A of *B. tabaci* Gennadius (Hemiptera: Aleyrodidae), originated from the Institute of Biotechnology and Germplasm Resources (IBGR), Yunnan Academy of Agricultural Sciences, Kunming City, China were used in this study. Adult *B. tabaci* was identified by the mitochondrial COI gene as cryptic species *B. tabaci* Q and A [48]. Whiteflies were cultured on a cotton plant, *Gossypium hirsutum* L. (Malvaceae) in screen cages (40 × 40 × 40 cm) inside a growth chamber separately with maintained conditions of 26 ± 0.5 °C, RH 60 ± 5%, and a photoperiod of 16 L:8D h at a light intensity of 1400–1725 lux [49]. Newly emerged (<48 h) adult whiteflies were used for all experimental bioassays.

### 2.2 Adult host selection and oviposition in multi-choice condition

Host selection of adults and their oviposition of both cryptic species *B. tabaci* Q and A were evaluated under multi-choice conditions using four tomato varieties, ANE, HF, HZ, YS, and two weed species, BGW and FM. According to Rodríguez-López et al. [50] with little modification, an apical leaflet of each tomato variety and one leaf of each weed species were placed randomly in a circle inside the screen cage (35 × 35 × 20 cm) (Fig. 1(B)). Each leaflet/leaf petiole was inserted in a single plastic-made transparent vial (3.5 cm diameter x 6 cm high with 2 cm opening) containing tap water. Leaflets/leaves were placed abaxial surface down with an angle of horizontal plane to the central direction of their tips, the centre formed with those of experimental leaflets/leaves (Fig. 1(C)). Thirty couples of newly emerged adults were collected in a plastic cup (4 cm diameter x 4.5 cm high) followed by cold treatment at 8 °C for 5 min to reduce their movement to facilitate handling and placed the uncovered cup at the centre of leaflets/leaves. The number of adults settled on the leaflets/leaves was counted at 24 h, 48 h and 72 h after initiation. The number of eggs laid by adults was counted on both abaxial and adaxial surfaces of leaflets/leaves after 72 h of adult infestation (Fig. 1(D)). Nine replications were carried out in this bioassay.

### 2.3 Immature development and survival rate

Five plants where one plant in one pot of each variety/species was used, and two leaves, 2nd and 3rd from the top, were selected from each plant. Sixty adults from each of two cryptic species *B. tabaci* Q and A were confined in clip-cage onto the abaxial leaf surfaces of each of the plant varieties/species (Fig. 1(D)). All adults with clip-cages were removed from the plant leaves 24 h after infestation. Eggs laid on abaxial surfaces were kept and twenty eggs were considered from each leaf for developmental study (from egg to adult eclosion) (Fig. 1(E)). All eggs on each leaf were monitored for survival (from egg to nymph, nymph to pupa, and pupa to adult) and calculated per cent survived (number of survived immature*100/total number of immature) immature of one leaf that considered one replication, making 10 replications in total (Fig. 1(F)). Immature was observed every day under a stereomicroscope until they developed into adults or died (Fig. 1(G and H)).

### 2.4 Adult survival and oviposition in seven days in no-choice condition

Adult survival and oviposition of both cryptic species *B. tabaci* Q and A on four tomato varieties and two weed species were assayed according to Di et al. [51] with some adjustments. Two plants of the same varieties/species were kept inside a 35 × 35 × 35 cm screen cage where two clip cages on each plant were attached to the abaxial surface of the 2nd and 3rd leaf from the top of the plant. The plants used were on a 10 cm diameter plastic pot. Five couples of newly emerged adults were released inside each clip cage and clip cages were removed from leaves after 6 h of adult infestation, adults were allowed to settle down on the leaf. Surviving adults were counted on plant leaves until 7 days of every 24 h after the adult was released. After 7 days, eggs laid on both abaxial and adaxial surfaces of the leaves were counted under a stereomicroscope (Fig. 1(G, H & I)). In this bioassay, eight replications, in total 96 plants (8 reps × 12 plants), were observed while one plant of two in one cage was considered one replication.

### 2.5 Leaf trichomes analysis

A square opening (0.5 cm × 0.5 cm) in a thin plastic transparent sheet was made to conduct the leaf trichomes analysis according to Hasanuzzaman et al. [52]. The opening with a plastic sheet was placed on the abaxial surface of the leaf lamina and trichomes exposed through the opening were identified as trichome categories [25,53,54] (Fig. S1) and were counted using a stereomicroscope (Fig. 1(I)). Data were taken from the middle position between the mid-vein and leaf margin and between the base and apex of the leaf lamina for all treatments. Eight replications, in total 48 plants (8 reps × 6 plants), were carried out for all treatments; where in each replication single mean of two values from each leaf was used and subjected to statistical analysis.

### 2.6 Data analysis

All observed data were analyzed by using SPSS statistical software (SPSS Inc., Chicago, IL, USA). Since the investigational whitefly species are two in number, we carried out an independent samples *t*-test to compare between species. The data of adult host selection, survival and oviposition, immature development and survival, and leaf trichomes densities were subjected to one-way ANOVA to test the significance of variances among the treatments of six plant species/varieties. Factorial ANOVA was applied to evaluate adult host selection and adult survival considered time a factor. The mean values were separated using the Least Significant Difference (LSD) Test at *P* ≤ 0.05 when values differed significantly among the treatments.

## 3 Results

### 3.1 Adult host selection and oviposition in multi-choice condition

**Fig. 2:**
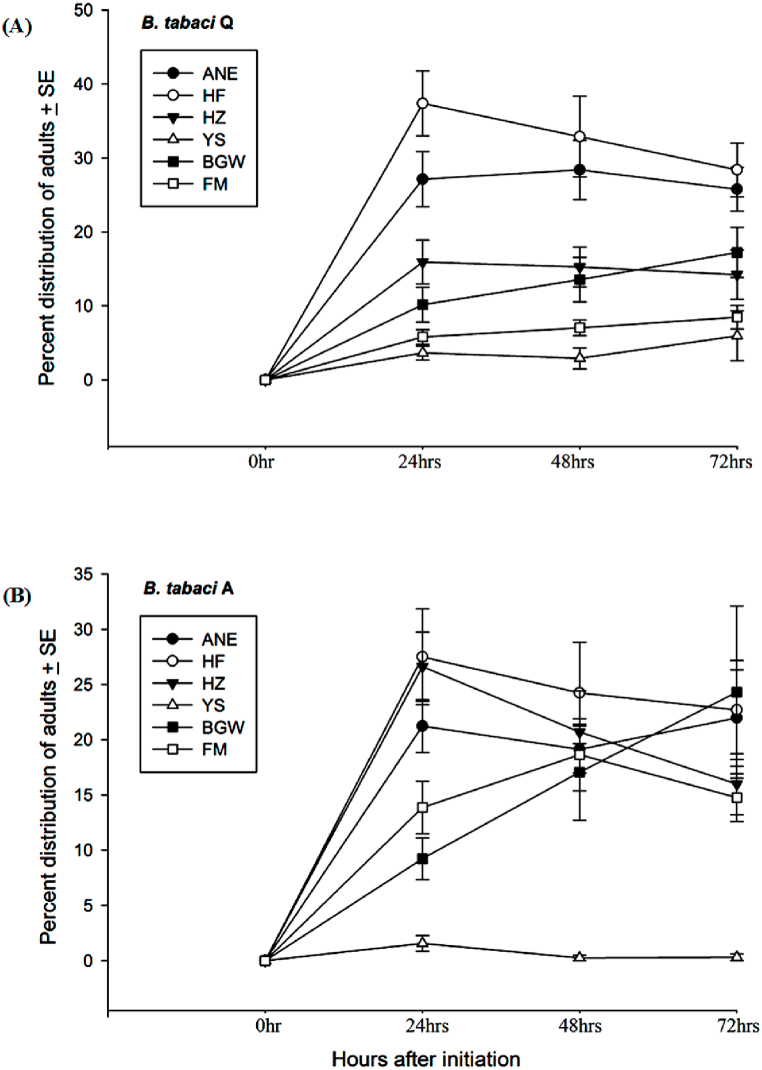
The distribution (%) (mean ± SE) of **(A)***B. tabaci* Q and **(B)***B. tabaci* A adults on the leaf of Ao-Ni-Er (ANE), He-Fen (HF), He-Zuo (HZ), Ye-Sheng (YS), Billy-Goat-Weed (BGW), and False-Mallow (FM). ±SE (standard error) was calculated for each treatment from three replications.

Adult host selection dynamics in the multi-choice condition of two cryptic species *B. tabaci* Q and A on different treatments of six plant species/varieties are shown in Fig. 2(A) and (B).

The significant interactions among whitefly species, plants and time (*F*_35,288_ = 8.396, *P* ≤ 0.001) were observed in host selection dynamics. The interaction effects between plant and time showed differences significantly (*F*_17,306_ = 14.157, *P* ≤ *0.001*). Host selection dynamics of *B. tabaci* Q showed significant differences at 24 h (*F*_5,53_ = 21.161, *P* ≤ *0.001*), 48 h (*F*_5,53_ = 12.788, *P* ≤ *0.001*) and 72 h (*F*_5,53_ = 8.407, *P* ≤ *0.001*) after adult infestation (Fig. 2 (A)). At 24 h, the highest percentage of adults was observed on He-Fen (37.36 ± 4.36%), medium-high on Ao-Ni-Er (27.13 ± 3.73%), medium-low on He-Zuo (15.91 ± 2.97%) and Billy-Goat-Weed (10.16 ± 2.36%), and the lowest on Ye-Sheng (3.63 ± 0.94%) and False-Mallow (5.78 ± 0.99%). At 48 h, the highest percentage of adults was found on He-Fen (32.88 ± 5.45%) and Ao-Ni-Er (28.39 ± 3.99%), medium of that on He-Zuo (15.26 ± 2.69%), Billy-Goat-Weed (13.54 ± 3.00%) and False-Mallow (7.02 ± 1.05%), where the lowest percentage value was on Ye-Sheng (2.90 ± 1.42%) (Fig. 2 (A)).

At 72 h, almost a similar trend was observed where the highest percentage of adults was counted on He-Fen (28.39 ± 3.63%) and Ao-Ni-Er (25.76 ± 2.96%), medium on He-Zuo (14.22 ± 3.32%) and Billy-Goat-Weed (17.22 ± 3.38%), where the lowest on Ye-Sheng (5.94 ± 3.37%) and False-Mallow (8.45 ± 1.58%). Adult host selection of *B. tabaci* A illustrated significant differences at 24 h (*F*_5,53_ = 14.476, *P* ≤ *0.001*), 48 h (*F*_5,53_ = 6.136, *P* ≤ *0.001*), and at 72 h (*F*_5,53_ = 4.219, *P* = 0.003) after adult infestation. At 24 h, the highest percentage of adults was observed on He-Fen (27.49 ± 4.32%), He-Zuo (26.61 ± 3.13%) and Ao-Ni-Er (21.23 ± 2.37%), the medium percentage was on False-Mallow (13.85 ± 2.37%) and Billy-Goat-Weed (9.22 ± 1.87%), and the lowest percentage was on Ye-Sheng (1.56 ± 0.71%) (Fig. 2 (B)). At 48 h, the highest percentage of adults was counted on He-Fen (24.23 ± 4.58%), He-Zuo (20.69 ± 3.72%), Ao-Ni-Er (19.12 ± 2.13%), False-Mallow (18.64 ± 3.26%), and Billy-Goat-Weed (17.06 ± 4.35%), where the lowest percentage was on Ye-Sheng (0.24 ± 0.24%). At 72 h, the highest percentage of adults was on Billy-Goat-Weed (24.30 ± 7.79%), He-Fen (22.69 ± 4.49%), Ao-Ni-Er (21.97 ± 4.35%), He-Zuo (15.97 ± 2.76%), False-Mallow (14.75 ± 2.17%), and the lowest percentage value was on Ye-Sheng (0.30 ± 0.30%).

The oviposition in multi-choice conditions was varied between *B. tabaci* Q and A cryptic species (*t*_106_ = 4.482, *P* ≤ *0.001*). Considering all six experimental host plants, *B. tabaci* Q laid more eggs (84.48 ± 11.01) than that of *B. tabaci* A (29.37 ± 5.47) (Table 1). The interaction effect between whitefly species and plants varied significantly (*F*_11,107_ = 9.646, *P* ≤ *0.001*). The number of eggs oviposited by *B. tabaci* Q varied (*F*_5,53_ = 7.744, *P* ≤ *0.001*) among the treatments where more eggs were laid on Ao-Ni-Er and He-Fen (Table 1). While the *B. tabaci* A oviposited more eggs on Billy-Goat-Weed and False-Mallow (*F*_5,53_ = 6.098, *P* ≤ *0.001*) among the six plants (Table 1).

**Table 1:** Total number of eggs (mean ± SE) laid by *B. tabaci* Q and A on the leaf of six experimental plants in multi-choice condition.

Experimental plants	Total number of eggs (mean ± SE)	
	*B. tabaci* Q	*B. tabaci* A
Ao-Ni-Er	171.7 ± 27.5 a	15.6 ± 2.6 b
He-Fen	146.2 ± 28.0 a	13.9 ± 4.1 b
He-Zuo	37.9 ± 12.2 b	18.1 ± 3.1 b
Ye-Sheng	41.2 ± 22.1 b	1.9 ± 1.8 b
Billy-Goat-Weed	57.7 ± 17.1 b	69.4 ± 23.1 a
False-Mallow	52.2 ± 13.9 b	57.3 ± 12.6 a

The means having the different letters in the same column differed significantly at *P* ≤ 0.05 (LSD_0.05_; Least Significant Difference at 5% level of probability).

### 3.2 Immature development duration of B. tabaci Q and A

**Fig. 3:**
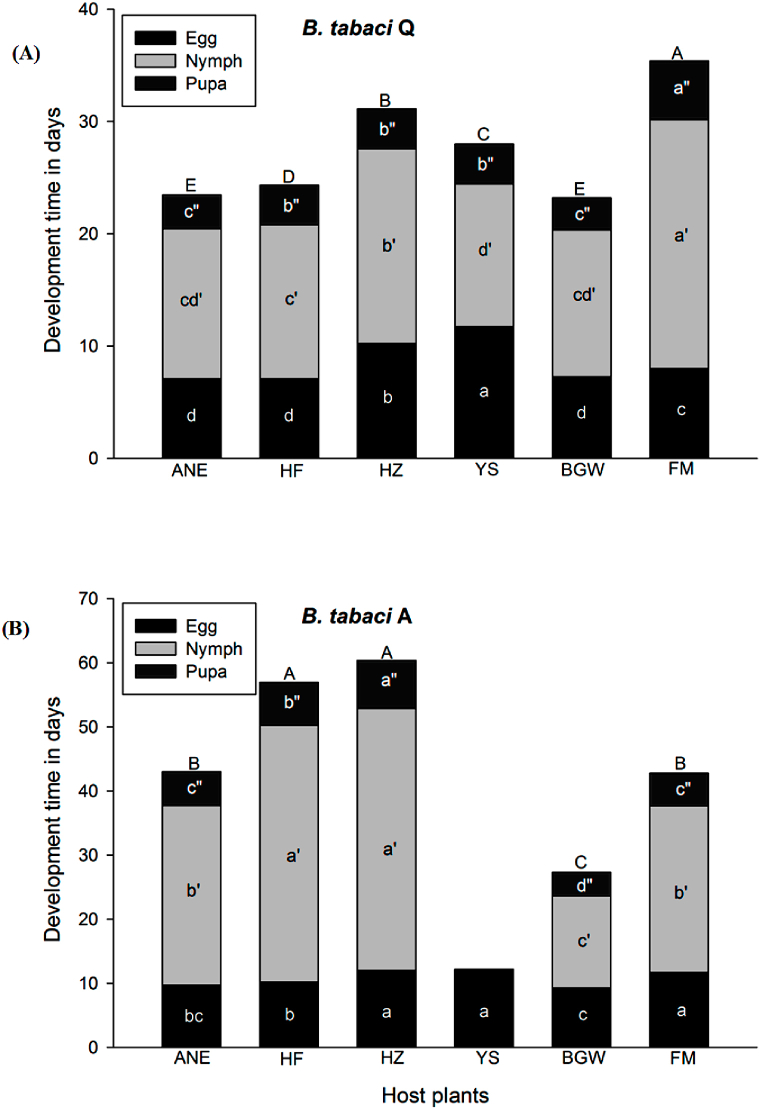
Immature development duration of (A) *B. tabaci* Q and (B) *B. tabaci* A cryptic species on Ao-Ni-Er (ANE), He-Fen (HF), He-Zuo (HZ), Ye-Sheng (YS), Billy-Goat-Weed (BGW), and False-Mallow (FM). The letters A, B, C, D, and E above the columns denoted significant differences in total immature development duration on plant species/varieties at *P* ≤ 0.05 (LSD_0.05_; Least Significant Difference). The letter on columns a, b, c; a', b', c'; and a", b", c", d" denoted significant differences in development duration among eggs, nymphs and pupae, respectively at *P* ≤ 0.05 (LSD_0.05_; Least Significant Difference at 5% level of probability).

The accumulated duration from egg to adult eclosion varied significantly (*t*_1402_ = −22.407, *P* ≤ *0.001*) between the cryptic species *B. tabaci* Q and A where the total duration for Q was 27.21 ± 0.16d and for A was 36.97 ± 0.56d (Fig. 3 (A) & (B)). The incubation period between *B. tabaci* Q and A did not differ (*t*_1834_ = −33.04, *P* = 0.249) on Ao-Ni-Er, He-Fen, He-Zuo, Ye-Sheng, Billy-Goat-Weed, and False-Mallow. While nymph development period varied significantly (*t*_1405_ = −18.326, *P* ≤ *0.001*) between them where nymphal duration for Q was 15.56 ± 0.12d and for A was 22.10 ± 0.47d. The pupal period also varied (*t*_1402_ = −16.664, *P* ≤ *0.001*) where Q was 3.58 ± 0.03d and A was 4.65 ± 0.06d. The interaction between whitefly species and plants showed differences for eggs (*F*_4,1403_ = 136.66, *P* ≤ *0.001*), for nymphs (*F*_4,1403_ = 493.19, *P* ≤ *0.001*), for pupae (*F*_4,1403_ = 245.44, *P* ≤ *0.001*) development and their cumulative development duration (*F*_4,1403_ = 513,99, *P* ≤ *0.001*). The egg incubation period of *B. tabaci* Q varied among the plants (*F*_5,1042_ = 1227.8, *P* ≤ *0.001*) where was shorter on Ao-Ni-Er (7.08 ± 0.02d), He-Fen (7.08 ± 0.02d), and Billy-Goat-Weed (7.25 ± 0.03d) and medium shorter on False-Mallow (7.99 ± 0.02d), medium length on He-Zuo (10.23 ± 0.04d), and longer on Ye-Sheng (11.74 ± 0.24d) (Fig. 3 (A) & (B)).

Development time of nymphs of *B. tabaci* Q differed among the plants (*F*_5,1009_ = 543.7, *P* ≤ *0.001*) and where nymph development duration was shorter on Ye-Sheng (12.70 ± 0.37d), medium shorter on Billy-Goat-Weed (13.08 ± 0.08d), Ao-Ni-Er (13.37 ± 0.08d), He-Fen (13.72 ± 0.07d), medium length on He-Zuo (17.34 ± 0.06d) and longer on False-Mallow (22.17 ± 0.32d) (Fig. 3). The development time of the pupal stage was also varied (*F*_5,1006_ = 325.0, *P* ≤ *0.001*), where the shortest on Billy-Goat-Weed (2.88 ± 0.02d) and Ao-Ni-Er (3.02 ± 0.04d) moderate on He-Fen (3.54 ± 0.04d), He-Zuo (3.57 ± 0.04d), and Ye-Sheng (3.55 ± 0.12d) and longest on False-Mallow (5.24 ± 0.07d) (Fig. 3 (A) & (B)). The cumulative development time of all immature varied (*F*_5,1006_ = 1004.07, *P* ≤ *0.001*) among the plant species and can be ranked from shortest to longest as Billy-Goat-Weed, Ao-Ni-Er <, He-Fen < Ye-Sheng < He-Zuo < False-Mallow (Fig. 3 (A) & (B)).

The egg incubation period of *B. tabaci* A varied among the plants (*F*_5,792_ = 227.4, *P* ≤ *0.001*) where shorter on Billy-Goat-Weed (9.25 ± 0.06d) and Ao-Ni-Er (9.73 ± 0.10d) and moderate on He-Fen (10.16 ± 0.06d) and longest on False-Mallow (11.69 ± 0.04d), He-Zuo (12.03 ± 0.12d), and Ye-Sheng (12.2 ± 0.13d) (Fig. 3 (A) & (B)). Development time of nymphs of *B. tabaci* A varied among the plants (*F*_4,396_ = 350.7, *P* ≤ *0.001*) where shorter time was on Billy-Goat-Weed (14.36 ± 0.09d), moderate on False-Mallow (25.99 ± 0.45d) and Ao-Ni-Er (28 ± 1.73d) and longer on He-Fen (40.05 ± 1.62d) and He-Zuo (40.85 ± 1.63d). Development time of the pupal stage also differed among plants (*F*_4,396_ = 280.4, *P* ≤ *0.001*) where shorter on Billy-Goat-Weed (3.68 ± 0.04d), medium shorter on False-Mallow (5.08 ± 0.05d) and Ao-Ni-Er (5.27 ± 0.19d), medium length on He-Fen (6.76 ± 0.23d), longer on He-Zuo (7.48 ± 0.13d) (Fig. 3 (A) & (B)). The cumulative developmental period of all immature varied greatly (*F*_4,396_ = 437.5, *P* ≤ *0.001*) among the plant species/varieties and can be ranked from shorter to longer as Billy-Goat-Weed < Ao-Ni-Er, False-Mallow < He-Fen, He-Zuo. All nymphs of *B. tabaci* A had died on Ye-Sheng consequently there were no pupae.

### 3.3 Immature survivorship of B. tabaci Q and A

**Fig. 4:**
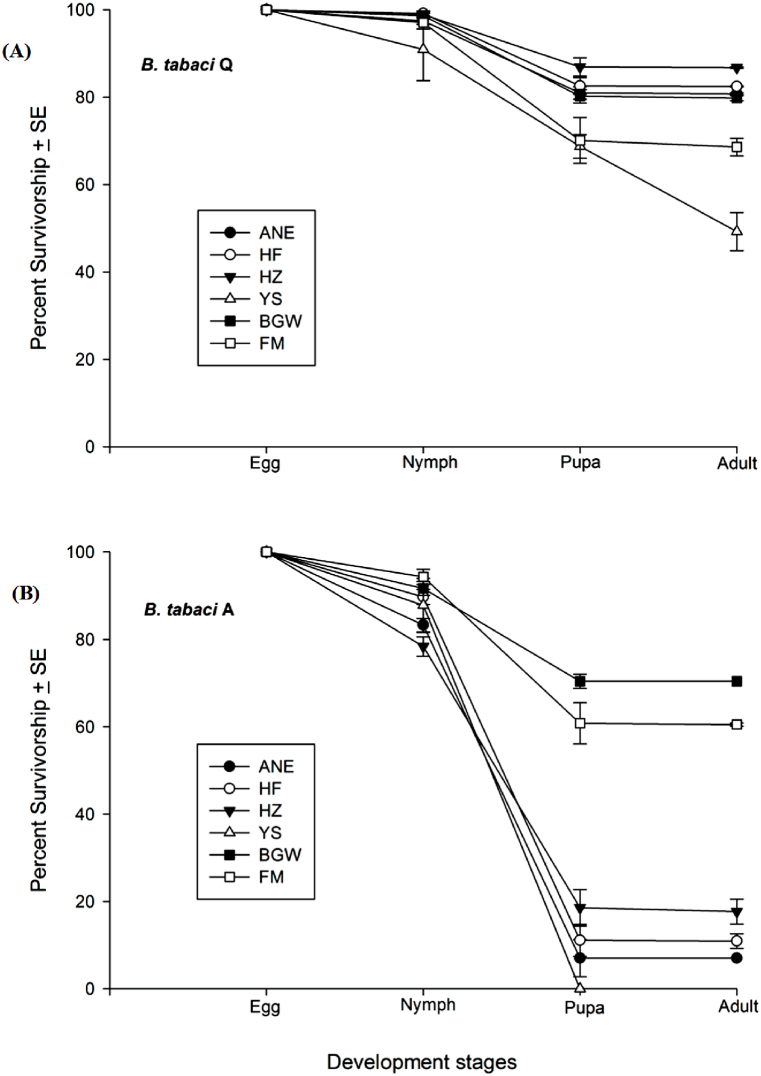
Survivorship/survival rate (%) (mean ± SE) of **(A)***B. tabaci* Q and **(B)***B. tabaci* A cryptic species on Ao-Ni-Er (ANE), He-Fen (HF), He-Zuo (HZ), Ye-Sheng (YS), Billy-Goat-Weed (BGW), and False-Mallow (FM). ±SE (standard error) was calculated for each treatment from three replications.

The egg survival rate varied significantly (*t*_102_ = 7.853, *P* ≤ *0.001*) between the two whitefly species where the percent survival of *B. tabaci* Q laid eggs were 97.72 **±** 0.66%, and *B. tabaci* A laid eggs about 87.83 ± 1.08%. The survival of nymphs also varied between whitefly species (*t*_102_ = 10.025, *P* ≤ *0.001*), where nymph survival of *B. tabaci* Q was 80.80 ± 1.37% and *B. tabaci* A was 35.29 ± 4.40% (Fig. 4 (A) & (B)). But in the case of pupa survival, there was no difference between whitefly species (*t*_88_ = −1.018, *P* = 0.084). The accumulated survival between Q and A varied significantly (*t*_102_ = 9.812, *P* ≤ *0.001*).

The interactions between whitefly species and plant varied significantly for eggs (*F*_10,79_ = 12.465, *P* ≤ *0.001*), nymph (*F*_10,79_ = 51.155, *P* ≤ *0.001*) and pupa (F_10,79_ = 5.052, *P* ≤ *0.001*). The survivorship of eggs laid by *B. tabaci* Q did not vary (*F*_5,53_ = 2.304, *P* = 0.059) among the six experimental plants (Fig. 4 (A) & (B)). The survival of nymphs varied significantly (*F*_5,53_ = 4.010, *P* = 0.004) among the plant species/varieties where a higher survival rate was on He-Zuo (87.76 ± 2.11%), He-Fen (83.28 ± 1.91%), Ao-Ni-Er (83.04 ± 1.45%), and Billy-Goat-Weed (81.21 ± 1.54%), moderate on Ye-Sheng (75.35 ± 2.69%), and lower of that was on False-Mallow (71.71 ± 5.23%) (Fig. 4 (A) & (B)). Pupal survival of *B. tabaci* Q varied (*F*_5,53_ = 6.318, *P* < 0.001) among the plant species/varieties where the lowest survival was observed on Ye-Sheng (65.92 ± 22.19%) and all most all pupae on other plants efficiently developed to adults (Fig. 4 (A) & (B)).

Eggs survivorship of *B. tabaci* A varied (*F*_5,50_ = 9.288, *P* ≤ *0.001*) among the plant species/varieties where the highest survival rate was observed on False-Mallow (94.311 ± 1.72%) and Billy-Goat-Weed (91.65 ± 1.58%), moderate on He-Fen (89.69 ± 1.73%) and Ye-Sheng (87.77 ± 6.18%), and lowest on He-Zuo (78.37 ± 2.22%) and Ao-Ni-Er (83.26 ± 1.52%) (Fig. 4). The survival of nymphs among the plant species/varieties varied greatly (*F*_5,50_ = 64.528, *P* ≤ *0.001*), the highest survival was observed on Billy-Goat-Weed (76.81 ± 1.60%) and False-Mallow (64.49 ± 4.71%), where only 8.19 ± 4.25% on Ao-Ni-Er, 12.14 ± 3.67% on He-Fen and 22.97 ± 4.18% on He-Zuo were survived, but all nymphs died on Ye-Sheng (Fig. 4 (A) & (B)). Pupal survival did not vary (*F*_4,36_ = 0.81, *P* = 0.527) among the plant species/varieties where all pupae successfully developed into adults except on Ye-Sheng, where there were no pupae.

### 3.4 Adult survival and oviposition in seven days in no-choice condition

**Fig. 5:**
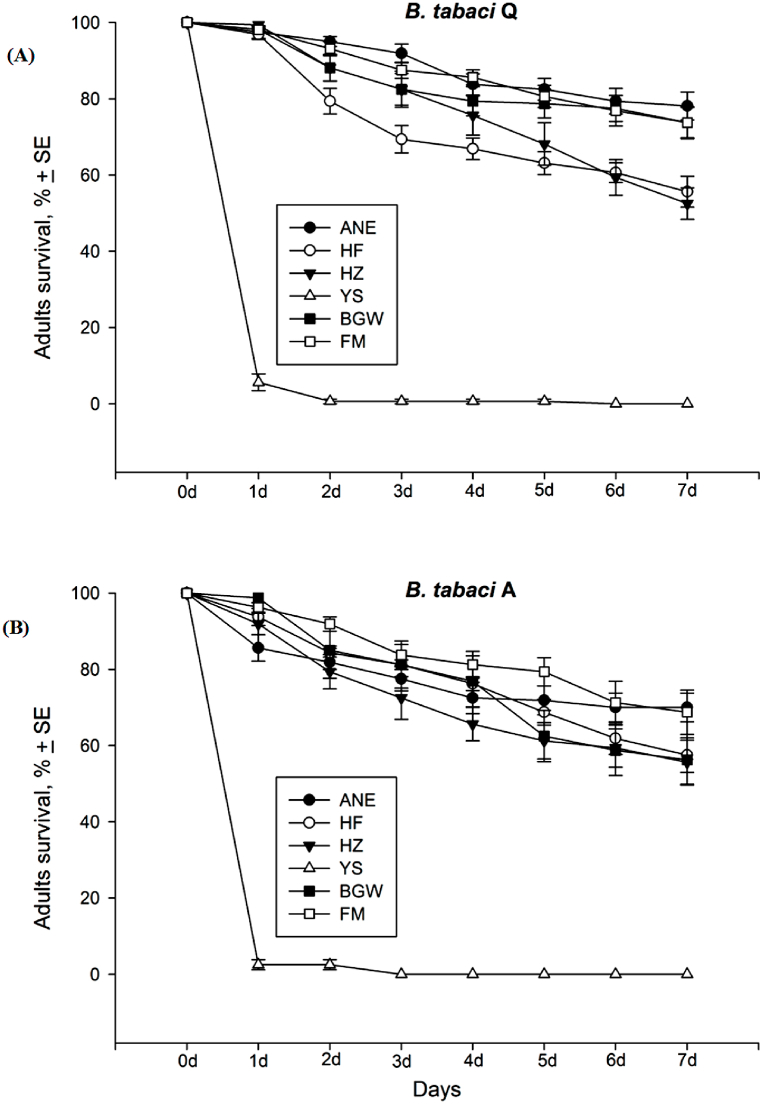
Adult survival (mean% ± SE) of **(A)***B. tabaci* Q and **(B)***B. tabaci* A cryptic species in seven days on Ao-Ni-Er (ANE), He-Fen (HF), He-Zuo (HZ), Ye-Sheng (YS), Billy-Goat-Weed (BGW), and False-Mallow (FM). ±SE (standard error) was calculated for each treatment from three replications.

The survival rate in seven days did not vary (*t*_670_ = 1.652, *P* = 0.994) between the cryptic species *B. tabaci* Q and A adults but the interaction among whitefly species, plants and days varied greatly (*F*_83,588_ = 75.150, *P* ≤ *0.001*). The interaction effects of survival rate varied between whitefly species and plants (*F*_11,671_ = 269.247, *P* ≤ *0.001*), whitefly species and days (*F*_13,658_ = 4.307, *P* ≤ *0.001*), and days and plants (*F*_41,630_ = 136.077, *P* ≤ *0.001*) (Fig. 5 (A) & (B)).

The survival rate of *B. tabaci* Q differed significantly in all seven days (*F*_5,47_ = 753.63, 194.70, 112.08, 102.97, 82.04, 81.46, 63.81; *P* ≤ 0.001, <0.001, <0.001, <0.001, <0.001, <0.001, <0.001) after adult infestation among the treatments of six plant species/varieties. On the first day after adult infestation, the survival rate on Ye-Sheng treatment was significantly lower (5.62 ± 2.2%) than that of other treatments (>96%). In the seven days after adult infestation, all adults died on Ye-Sheng treatment where 55.62 ± 4.05% and 52.50 ± 4.11% survival were observed on He-Fen and He-Zuo treatments, respectively were lower than those of Ao-Ni-Er (78.12 ± 3.65%), Billy-Goat-Weed (73.75 ± 4.19%) and False-Mallow (73.75 ± 3.98%) treatments (Fig. 5 (A) & (B)).

The survival rate of *B. tabaci* A also varied significantly in seven days (*F*_5,47_ = 278.29, 98.38, 71.92, 59.33, 46.22, 32.33, 27.61; *P* = < 0.001, <0.001, <0.001, <0.001, <0.001, <0.001, <0.001) after adult introduction among the six treatments. On the first day after adult introduction, only 2.5 ± 1.33% of adults survived on Ye-Sheng treatment which was significantly lower than those of all other treatments. In the seven days after adult introduction, all adults died on Ye-Sheng, where 70.00 ± 3.77%, 57.50 ± 4.53%, 55.62 ± 5.78%, 56.25 ± 6.66% and 68.75 ± 5.80% survival were observed on Ao-Ni-Er, He-Fen, He-Zuo, Billy-Goat-Weed and False-Mallow, respectively (Fig. 5 (A) & (B)).

The total number of eggs laid in seven days varied between the cryptic species of *B. tabaci* Q and A (*t*_94_ = 2.512, *P* = 0.010) where more number of eggs laid by Q (200.79 ± 18.15) than the number of eggs laid by A (146.04 ± 12.06). The interaction effects of ovipositional performance between whitefly species and plants also differed (*F*_11,95_ = 16.788, *P* ≤ *0.001*). The total number of eggs laid by *B. tabaci* Q (*F*_5,47_ = 14.618, *P* ≤ *0.001*) and A (*F*_5,47_ = 23.565, *P* ≤ *0.001*) varied in seven days on the experimental six plants (Table 2).

**Table 2:** Total number of eggs (mean ± SE) oviposited by *B. tabaci* Q and A on Ao-Ni-Er (ANE), He-Fen (HF), He-Zuo (HZ), Ye-Sheng (YS), Billy-Goat-Weed (BGW), and False-Mallow (FM) in seven days.

Experimental plants	Total number of eggs (mean ± SE)	
	*B. tabaci* Q	*B. tabaci* A
Ao-Ni-Er	299.4 ± 19.8 a	208.5 ± 15.6 a
He-Fen	226.4 ± 46.4 ab	180.1 ± 19.9 ab
He-Zuo	213.7 ± 28.1 b	121.1 ± 11.0 c
Ye-Sheng	7.1 ± 2.7 c	2.3 ± 1.1 d
Billy-Goat-Weed	160.6 ± 19.4 b	156.9 ± 16.6 BCE
False-Mallow	297.5 ± 33.5 a	207.4 ± 22.4 a

This means having the different letters in the same column differed significantly at *P* ≤ 0.05 (LSD_0.05_; Least Significant Difference at 5% level of probability).

### 3.5 Leaf trichomes analysis

In the experimental plants, four types of leaf trichomes were identified Type III, Type IV, Type V and Type VI. The density in a unit area of all trichomes varied among the plants as Type III (*F*_5,47_ = 12.857, *P* ≤ *0.001*), Type IV (*F*_5,47_ = 244.478, *P* ≤ *0.001*), Type V (*F*_5,47_ = 20.454, *P* ≤ *0.001*) and Type VI (*F*_5,47_ = 4.493, *P* = 0.002) (Table 3).

**Table 3:** Densities (mean ± SE in number) of leaf trichomes per 0.25 cm^2^ leaf area of Ao-Ni-Er (ANE), He-Fen (HF), He-Zuo (HZ), Ye-Sheng (YS), Billy-Goat-Weed (BGW), and False-Mallow (FM).

Experimental plants	Type III	Type IV	Type V	Type VI
Ao-Ni-Er	5.56 ± 0.6 b	1.68 ± 0.6 b	104.56 ± 13.3 a	9.87 ± 1.5 b
He-Fen	3.81 ± 0.8 bce	0.0 ± 0.0 b	55.06 ± 11.7 b	15.63 ± 2.7 ab
He-Zuo	4.19 ± 0.9 bce	7.37 ± 4.5 b	66.5 ± 15.6 b	29.06 ± 12.3 a
Ye-Sheng	0.0 ± 0.0 d	272.44 ± 16.7 a	0.0 ± 0.0 c	6.75 ± 1.3 b
Billy-Goat-Weed	8.18 ± 1.1 a	0.0 ± 0.0 b	5.00 ± 0.5 c	0.0 ± 0.0 b
False-Mallow	3.19 ± 0.5 c	0.0 ± 0.0 b	0.0 ± 0.0 c	0.0 ± 0.0 b

This means having the different letters in the same column differed significantly at *P* ≤ 0.05 (LSD_0.05_; Least Significant Difference at 5% level of probability).

## 4 Discussion

Host selection is an important behavioural activity of insects such as whiteflies to find their preferred host plants for settling, feeding, and oviposition. In field conditions, the whitefly species affect their host selection on different varieties of tomato as well as weed species nearby the crops. Our data of host selection dynamics and oviposition showed significant variation among the four tomato varieties and two weed species of both cryptic species *B. tabaci* Q and A.

The host preference trend of the cryptic species *B. tabaci* Q at all three observational times, 24 h, 48 h, and 72 h was similar where they mostly preferred tomato varieties, He-Fen and Ao-Ni-Er, moderately He-Zuo and Billy-Goat-Weed over False-Mallow and Ye-Sheng. The *B. tabaci* A, at the earlier time mostly settled on tomato varieties but later on resettled evenly on tomato varieties and weed species except for Ye-Sheng. Interestingly, *B. tabaci* Q laid more eggs on tomato varieties Ao-Ni-Er and He-Fen, and *B. tabaci* A laid more eggs on weed species, Billy-Goat-Weed and False-Mallow. Ao-Ni-Er and He-Fen showed faster growth, flat leaves, and more number of trichome Type V which are the foremost reasons for adult attraction and oviposition [[55], [56], [57], [58], [59]]. On the contrary, *B. tabaci* A was deterred from tomatoes and was influenced by the weed species for settling and oviposition.

Fewer numbers of Q and A adults settled on the tomato variety, He-Zuo, and the weed species, False-Mallow, which might be due to leaf size, leaf colour, leaf shape, and trichomes [52,56,60,61]. However, due to the presence of glandular trichomes and leaf lamina shape, they refused Ye-Sheng and He-Zuo [25,50,55]. In the study, we observed that the leaf lamina of He-Zuo was skew in shape with more glandular trichome type VI and Ye-Sheng leaf lamina was flat in shape having more glandular trichome type IV. These properties deter whiteflies from He-Zuo and Ye-Sheng tomato varieties. The results of this study were harmonic with previous studies that stated the presence of glandular trichome type IV and VI in *Solanum* spp. Showed a high level of host resistance to insect species likely aphids and whiteflies [55,62]. The presence of these glandular trichomes types IV and VI secret acyl-sugar and toxic phenolic compounds that mediate tomato plants' resistance to many whiteflies [25,56,63,64].

The immature developmental period is related to feeding preference, antibiosis, and nutritional composition in host plants and insect species [43,47,65,66]. The development time of *B. tabaci* Q was shorter than *B. tabaci* A which indicates Q is more invasive than A. The egg incubation of both *B. tabaci* Q and A was faster on Ao-Ni-Er, He-Fen, and Billy-Goat-Weed than that of other plant species/varieties. The nymph and pupa of *B. tabaci* Q developed faster on all tested plants either tomato varieties or weed species. The nymph and pupa of *B. tabaci* A were developed faster on Billy-Goat-Weed and False-Mallow than tomato varieties. These results illustrated that the preferred hosts for Q are tomato varieties and Billy-Goat-Weed but for A are mostly weed species. Though the host plant is considered the main factor for influencing the immature development of whiteflies [47], but whitefly cryptic species are also an influencing object for host selection [18,43,67].

The egg and nymph survival varied between *B. tabaci* Q and A on six experimental plants where more egg and nymph of *B. tabaci* Q were survived [43] than the egg and nymph of *B. tabaci* A. This result again confirms that *B. tabaci* Q is more invasive than *B. tabaci* A. The nymph survival rate of *B. tabaci* Q was higher on Ao-Ni-Er, He-Fen, He-Zuo, and Billy-Goat-Weed than Ye-Sheng and False-Mallow. The egg and nymph survival rate of *B. tabaci* A was higher on Billy-Goat-Weed and False-Mallow than tomato varieties, where only 0–27% of nymphs survived on tomato varieties. This result suggests that tomatoes are unsuitable host plants for *B. tabaci* A. Almost all pupae of both *B. tabaci* Q and A successfully developed into adults in all bioassays [47] except for Ye-Sheng.

Like many other herbivorous insect species, the adult survival of different *B. tabaci* populations or species are impacted by host plant species [51,68]. In this study reported herein, the interaction effect of *B. tabaci* Q and A adult survival and oviposition in seven days on six experimental plants varied greatly. Adults of both species survived a very short time on Ye-Sheng and oviposited very few eggs where after 24 h almost all adults died. This is due to the presence of a high number of glandular trichome Type IV on Ye-Sheng leaves [58,65,69,70]. During experimentation, it was found that whitefly adults entrapped in sticky exudates released from glandular trichomes type IV and subsequently died [70,71]. Nonetheless, their survival rate and oviposition differed from other tomato varieties and weed species and the rate of survival in tomatoes supports the earlier results described by Di et al. [51].

Results of development duration, survival rate, and oviposition are suggesting that the adaptation capacity of *B. tabaci* Q is wider regarding plant species than that of *B. tabaci* A. These results support previously studied reports [43,[72], [73], [74], [75]]. This higher capacity, adaptation to a wider range of plant species/varieties of *B. tabaci* Q [41,43,75,76] may have a chance to replace other cryptic species like A.

Our results illustrate that both weed species, Billy-Goat-Weed and False-Mallow are good hosts of whiteflies and act as a source of whiteflies. Though fewer adults settled on False-Mallow in multi-choice conditions the results, adult longevity and oviposition in no-choice conditions revealed that False-Mallow is a good host of whiteflies. During multi-choice bioassay, few adults settled on False-Mallow might be due to leaf size, shape, leaf colour, odour, and trichome type and densities [52,56,60,61] or sometimes conflicting results obtained due to various host plant species/varieties [77,78]. However, whiteflies can overwinter on these weeds in cropping off-season and even get shelter when insecticides are applied on the main crop fields. Furthermore, these two weed species are a reservoir of many viruses [37,38,[79], [80], [81], [82]] that are transmitted by whiteflies to tomatoes or other crop species. Based on the result described herein, weeds need to be considered in plant protection activities, especially in field crops.

In this study, we observed four types (Fig. 1(S)) [53,54] of trichomes e.g. Type III, Type IV, Type V, and Type VI on the leaves of tomatoes and weeds. Type III is non-glandular with 4–8 cells, and 0.4–1.0 mm long, Type IV is glandular with unicellular, and 0.2–0.4 mm long, Type V is non-glandular with unicellular, and 0.2–0.4 mm long; and Type VI is glandular, less than 0.2 mm long, head bears 4 secretory cells [25,53,54]. We observed that plants bearing more glandular trichomes had less number of non-glandular trichomes and support with the described result of Oriani and Vendramim [55]. Depending on trichome types and densities on the leaves of tomatoes and weeds whiteflies are impacted differently.

## 5 Conclusion

The findings state that both plants and whiteflies mediate their host selection, oviposition, development, and survival. Tomatoes are unsuitable hosts for *B. tabaci* A as prolonged development duration, high mortality of nymph, and rejection by the adults during host selection. Comparative performances between *B. tabaci* Q and A cryptic species illustrate Q-dominated A. Immature performances do not dictate adult host selection dynamics. Weeds can play a significant role to breed both *B. tabaci* Q and A may be employed as sources of whiteflies and generate hazardous for tomato cultivation. Finally, it is suggested that leaf glandular trichomes may be targeted in plant breeding programmes to develop resistant tomato varieties against whiteflies.

## Author contributions

Conceived and designed the experiments; Md. Mostafizur Rahman Shah, Zhongkai Zhang and Jian Hu. Performed the experiments; Md. Mostafizur Rahman Shah, Zhongkai Zhang and Jian Hu. Analyzed and interpreted the data; Md. Mostafizur Rahman Shah, Ahmed Gaber and Akbar Hossain. Contributed reagents, materials, analysis tools or data; Md. Mostafizur Rahman Shah, Zhongkai Zhang and Jian Hu. Wrote the paper: Md. Mostafizur Rahman Shah, Zhongkai Zhang, Jian Hu, Ahmed Gaber and Akbar Hossain. All authors have read and agreed to the published version of the manuscript in Heliyon.

## Funding

This research work was supported by the 10.13039/501100004573Ministry of Agriculture Key Lab of Southwestern Crop Gene Resources and Germplasm Innovation, Yunnan Provincial Key Lab of Agricultural Biotechnology, Biotechnology and Germplasm Resources Institute, Yunnan Academy of Agricultural Sciences, 650,223 Kunming, China. The researchers would like to acknowledge the Deanship of Scientific Research, 10.13039/501100006261Taif University, Taif, Saudi Arabia for funding this work.

## Institutional review board statement

Not applicable.

## Informed consent statement

Not applicable.

## Data availability statement

Most of the data are available in all Tables and Figures of the manuscripts.

## Declaration of competing interest

The authors declare that they have no known competing financial interests or personal relationships that could have appeared to influence the work reported in this paper.

## References

[1] De Barro P.J. (1995). https://www.cabdirect.org/cabdirect/abstract/19961104915.

[2] Jones D.R. (2003). Plant viruses transmitted by whiteflies. Eur. J. Plant Pathol..

[3] Liu T.X. (2007). Life history of *Eretmocerus melanoscutus* (Hymenoptera: Aphelinidae) parasitizing nymphs of *Bemisia tabaci* biotype B (Homoptera: Aleyrodidae). Biol. Control.

[4] Bayhan E., Ulusoy M.R., Brown J.K. (2006). Host range, distribution, and natural enemies of *Bemisia tabaci* ‘B biotype’ (Hemiptera: Aleyrodidae) in Turkey. J. Pest. Sci..

[5] Bedford I.D., Markham P.G., Brown J.K., Rosell R.C. (1994). Geminivirus transmission and biological characterization of whitefly (*Bemisia tabaci*) biotypes from different world regions. Ann. Appl. Biol..

[6] Mound L.A., Halsey S.H. (1978). https://www.cabdirect.org/cabdirect/abstract/19780557087.

[7] Chou I. (1949). Listo de la konataj Aleurodoj “Homopteroj” en Cinio. J. Chin. Entomol..

[8] Zhang L.P., Zhang Y.J., Zhang W.J., Wu Q.J., Xu B.Y., Chu D. (2005). Analysis of genetic diversity among different geographical populations and determination of biotypes of *Bemisia tabaci* in China. J. Appl. Entomol..

[9] Chu D., Zhang Y.J., Brown J.K., Cong B., Xu B.Y., Wu Q.J., Zhu G.R. (2006). The introduction of the exotic Q biotype of *Bemisia tabaci* from the Mediterranean region into China on ornamental crops. Fla. Entomol..

[10] Wu X., Li Z., Hu D., Shen Z. (2003). Identification of Chinese populations of *Bemisia tabaci* (Gennadius) by analyzing ribosomal ITS1 sequence. Prog. Nat. Sci..

[11] De Barro P.J., Trueman W.H., Frohlich D.R. (2005). *Bemisia argentifolii* is a race of *B. tabaci* (Hemiptera: Aleyrodidae): the molecular genetic differentiation of B. tabaci populations around the world. Bull. Entomol. Res..

[12] Li S.J., Xue X., Ahmed M.Z., Ren S.X., Du Y.Z., Wu J.H., Cuthbertson A.G., Qiu B.L. (2011). Host plants and natural enemies of *Bemisia tabaci* (Hemiptera: Aleyrodidae) in China. Insect Sci..

[13] Berlinger M.J. (1986). Host plant resistance to *Bemisia tabaci*. Agric. Ecosyst. Environ..

[14] Costa H.S., Brown J.K. (1991). Variation in biological characteristics and esterase patterns among populations of *Bemisia tabaci*, and the association of one population with silverleaf symptom induction. Entomol. Exp. Appl..

[15] Cohen S., Duffus J.E., Liu H.Y. (1992). A new *Bemisia tabaci* biotype in the southwestern United States and its role in silverleaf of squash and transmission of lettuce infectious yellows virus. Phytopathology.

[16] Martin J.H., Mifsud D., Rapisarda C. (2000). The whiteflies (Hemiptera: Aleyrodidae) of europe and the mediterranean basin. Bull. Entomol. Res..

[17] Perring T.M. (2001). The *Bemisia tabaci* species complex. Crop Protect..

[18] Milenovic M., Wosula E.N., Rapisarda C., Legg J.P. (2019). Impact of host plant species and whitefly species on feeding behavior of *Bemisia tabaci*. Front. Plant Sci..

[19] Bedford I.D., Briddon R.W., Markham P.G. (1992). Bemisia tabaci-biotype characterization and the threat of this whitefly species to agriculture, Proceedings. British Crop Protection Conference-Pests and Diseases.

[20] Sánchez-Campos S., Navas-Castillo J., Camero R., Soria C., Díaz J.A., Moriones E. (1999). Displacement of tomato yellow leaf curl virus (TYLCV)-Sr by TYLCV-Is in tomato epidemics in Spain. Phytopathology.

[21] Abdullahi I., Winter S., Atiri G.I., Thottappilly G. (2003). Molecular characterization of whiteﬂy, *Bemisia tabaci* (Hemiptera: Aleyrodidae) populations infesting cassava. Bull. Entomol. Res..

[22] Cohen S., Antignus Y., Harris K.F. (1994). Advances in Disease Vector Research.

[23] Engene N., Rottacker E.C., Kaštovský J., Byrum T., Choi H., Ellisman M.H., Komárek J., Gerwick W.H. (2012). Moorea producens gen. nov., sp. nov. and Moorea bouillonii comb. nov., tropical marine cyanobacteria rich in bioactive secondary metabolites. Int. J. Syst. Evol. Microbiol..

[24] Kortbeek R.W., Xu J., Ramirez A., Spyropoulou E., Diergaarde P., Otten-Bruggeman I., de Both M., Nagel R., Schmidt A., Schuurink R.C., Bleeker P.M. (2016). Engineering of tomato glandular trichomes for the production of specialized metabolites. Methods Enzymol..

[25] Glas J.J., Schimmel B.C., Alba J.M., Escobar-Bravo R., Schuurink R.C., Kant M.R. (2012). Plant glandular trichomes as targets for breeding or engineering of resistance to herbivores. Int. J. Mol. Sci..

[26] Schilmiller A.L., Last R.L., Pichersky E. (2008). Harnessing plant trichome biochemistry for the production of useful compounds. Plant J..

[27] Simmons A.T., Gurr G.M. (2005). Trichomes of *Lycopersicon* species and their hybrids: effects on pests and natural enemies. Agric. For. Entomol..

[28] Weinhold A., Baldwin I.T. (2011). Trichome-derived O-acyl sugars are a first meal for caterpillars that tags them for predation. Proc. Natl. Acad. Sci. USA.

[29] Norris R.F., Kogan M. (2000). Interactions between weeds, arthropod pests, and their natural enemies in managed ecosystems. Weed Sci..

[30] Capinera J.L. (2005). Relationships between insect pests and weeds: an evolutionary perspective. Weed Sci..

[31] Capinera J.L. (2001).

[32] Tan P.H.N., Wong S.M., Wu M., Bedford I.D., Saunders K., Stanley J. (1995). Genome organization of ageratum yellow vein virus, a monopartite whitefly-transmitted geminivirus isolated from a common weed. J. Gen. Virol..

[33] Roye M.E., McLaughlin W.A., Nakhla M.K., Maxwell D.P. (1997). Genetic diversity among geminiviruses associated with the weed species *Sida* sp., *Macroptilium lathyroides* and *Wissadula amplissima* from Jamaica. Plant Dis..

[34] Sanz A.I., Fraile A., Garcia-Arenal F., Zhou X., Robinson D.J. (2000). Multiple infection recombination and genome relationships among begomovirus isolates found in cotton and other plants in Pakistan. J. Gen. Virol..

[35] Konate G., Barro N., Fargette D., Swanson M.M., Harrison B.D. (1995). Occurrence of whitefly-transmitted geminiviruses in crops in Burkina Faso and their serological detection and differentiation. Ann. Appl. Biol..

[36] Fauquet C.M., Briddon R.W., Brown J.K., Moriones B., Stanley J., Zerbini M., Zhou X. (2008). Geminivirus strain demarcation and nomenclature. Arch. Virol..

[37] Liu P., Xie Y., Zhou X.P. (2009). *Malvastrum coromandelianum* is an alternative host of Tomato yellow leaf curl China virus. Plant Pathol..

[38] Wu J., Mugiira R.B., Zhou X. (2007). *Malvastrum* leaf curl Guangdong virus is a distinct monopartite begomovirus. Plant Pathol..

[39] Nombela G., Beitia F., Muñiz M. (2003). A differential interaction study of *Bemisia tabaci* Q‐biotype on commercial tomato varieties with or without the *Mi* resistance gene, and comparative host responses with the B‐biotype. Entomol. Exp. Appl..

[40] Matsuura S., Hoshino S. (2009). Effect of tomato yellow leaf curl disease on reproduction of *Bemisia tabaci* Q biotype (Hemiptera: Aleyrodidae) on tomato plants. Appl. Entomol. Zool..

[41] Pan H., Chu D., Ge D., Wang S., Wu Q., Xie W., Jiao X., Liu B., Yang X., Yang N., Su Q. (2011). Further spread of and domination by *Bemisia tabaci* (Hemiptera: Aleyrodidae) biotype Q on field crops in China. J. Econ. Entomol..

[42] Cuthbertson A.G.S., Buxton J.H., Blackburn L.F., Mathers J.J., Robinson K.A., Powell M.E., Fleming D.A., Bell H.A. (2012). Eradicating *Bemisia tabaci* Q biotype on poinsettia plants in the UK. Crop Protect..

[43] Jiao X., Xie W., Wang S., Wu Q., Zhou L., Pan H., Liu B., Zhang Y. (2012). Host preference and nymph performance of B and Q putative species of *Bemisia tabaci* on three host plants. J. Pest. Sci..

[44] Shi X., Pan H., Zhang H., Jiao X., Xie W., Wu Q., Wang S., Fang Y., Chen G., Zhou X., Zhang Y. (2014). *Bemisia tabaci* Q carrying tomato yellow leaf curl virus strongly suppresses host plant defenses. Sci. Rep..

[45] McKenzie C.L., Osborne L.S. (2017). *Bemisia tabaci* MED (Q biotype) (Hemiptera: Aleyrodidae) in Florida is on the move to residential landscapes and may impact open-field agriculture. Fla. Entomol..

[46] Yao F.L., Zheng Y., Huang X.Y., Ding X.L., Zhao J.W., Desneux N., He Y.X., Weng Q.Y. (2017). Dynamics of *Bemisia tabaci* biotypes and insecticide resistance in Fujian province in China during 2005-2014. Sci. Rep..

[47] Shah M.M.R., Liu T.X. (2013). Feeding experience of *Bemisia tabaci* (Hemiptera: Aleyrodidae) affects their performance on different host plants. PLoS One.

[48] Frohlich D.R., Torres-Jerez I., Bedford I.D., Markham P.G., Brown J.K. (1999). A phylogeographical analysis of *Bemisia tabaci* species complex based on mitochondrial DNA markers. Mol. Ecol..

[49] Greenberg S.M., Jones W.A., Liu T.X. (2002). Interactions among two species of *Eretmocerous* (Hymenoptera: Aphelidae), two species of Whiteflies (Homoptera: Aleyrodidae), and tomato. Environ. Entomol..

[50] Rodríguez-López M.J., Garzo E., Bonani J.P., Fernandez-Munoz R., Moriones E. (2012). Acylsucrose-producing tomato plants forces *Bemisia tabaci* to shift its preferred settling and feeding site. PLoS One.

[51] Di N., Zhang K., Zhang F., Wang S., Liu T.X. (2018). Polyculture and monoculture affect the fitness, behavior and detoxification metabolism of *Bemisia tabaci* (Hemiptera: Aleyrodidae). Front. Physiol..

[52] Hasanuzzaman A.T.M., Islam M.N., Zhang Y., Zhang C.Y., Liu T.X. (2016). Leaf morphological characters can be a factor for intra-varietal preference of whitefly *Bemisia tabaci* (Hemiptera: Aleyrodidae) among eggplant varieties. PLoS One.

[53] Channarayappa S.G., Muniyappa V., Frist R.H. (1992). Resistance of *Lycopersicon* species to *Bemisia tabaci*, a tomato leaf curl virus vector. Can. J. Bot..

[54] Luckwill L.C. (1943). The genus *Lycopersicon*: a historical, biological and taxonomic survey of the wild and cultivated tomato. Aberd. Univ. Stud..

[55] Oriani M.A.D., Vendramim J.D. (2010). Influence of trichomes on attractiveness and ovipositional preference of *Bemisia tabaci* (Genn.) B biotype (Hemiptera: Aleroydae) on tomato genotypes. Neotrop. Entomol..

[56] Heinz K.M., Zalom F.G. (1995). Variation in trichome based resistance to *Bemisia argentifolii* (Homoptera: Aleyrodidae) oviposition on tomato. J. Econ. Entomol..

[57] Snyder J.C., Simmons A.M., Thacker R.R. (1998). Attractancy and ovipositional response of adult *Bemisia argentifolii* (Homoptera: Aleyrodidae) to type IV trichome density on leaves of *Lycopersicon hirsutum* grown in three day-length regimes. J. Entomol. Sci..

[58] Toscano L.C., Boiça A.L., Maruyama W.I. (2002). Nonpreference of whitefly for oviposition in tomato genotypes. Sci. Agric..

[59] Fancelli M., Vendramim J.D., Frighetto R.T.S., Lourencao A.L. (2005). Glandular exudate of tomato genotypes and development of *B. tabaci* (Genn.) (Sternorryncha: Aleyrodidae) biotype B, Neotrop. Entomologiste (Paris).

[60] Ozgur A.F., Sckeroglu E. (1986). Population development of *Bemisia tabaci* (Homoptera: Aleyrodidae) on various cotton varieties in Cukurova, Turkey. Agric. Ecosyst. Environ..

[61] Kishaba A.N., Castle S., McCreight J.D., Desjardins P.R. (1992). Resistance of white-flowered gourd to sweet potato whitefly. Hortic. Sci. (Stuttg.).

[62] Goffreda J.C., Mutschler M.A. (1989). Inheritance of potato aphid resistance in hybrids between *Lycopersicon esculentum* and *Lycopersicon pennellii*. Theor. Appl. Genet..

[63] Hartman J.B., St Clair D.A. (1999). Variation for aphid resistance and insecticidal acyl sugar expression among and within *Lycopersicon pennellii*-derived inbred backcross lines of tomato and their F_1_ progeny. Plant Breed..

[64] Maluf R.W., Maciel G.M., Gomes L.A.A., Cardoso M.G. (2010). Broad-spectrum arthropod resistance in hybrids between high- and low-acylsugar tomato lines. Crop Sci..

[65] Baldin E.L.L., Vendramim J.D., Lourencão A.L. (2005). Resistance of tomato genotypes to the whitefly *Bemisia tabaci* (Gennadius) biotype B (Hemiptera: Aleyrodidae), neotrop. Entomologiste (Paris).

[66] Liu Y.H., Kang Z.W., Guo Y., Zhu G.S., Shah M.M.R., Song Y., Fan Y.L., Jing X.F., Liu T.X. (2016). Nitrogen hurdle of host alternation for a polyphagous aphid and the associated changes of endosymbionts. Sci. Rep..

[67] Jiang Y.X., Lei H., Collar J.L., Martin B., Muñiz M., Fereres A. (1999). Probing and feeding behavior of two distinct biotypes of *Bemisia tabaci* (Homoptera: Aleyrodidae) on tomato plants. J. Econ. Entomol..

[68] Zhang K., Di N., Ridsdill S.J., Zhang B.W., Tan X.L., Cao H.H. (2014). Does a multi-plant diet benefit a polyphagous herbivore? A case study with *Bemisia tabaci*. Entomol. Exp. Appl..

[69] Fancelli M., Vendramim J.D. (2002). Development of *Bemisia tabaci* (Gennadius, 1889) biotype B on *lycopersicon* spp. genotypes. Sci. Agric..

[70] Fancelli M., Vendramim J.D., Lourenção A.L., Dias C.T.S. (2003). Atratividade e preferência para oviposição de *Bemisia tabaci* (Gennadius) (Hemiptera: Aleyrodidae) biótipo B em genótipos de tomateiro. Neotrop. Entomol..

[71] Muigai S.G., Schuster D.J., Bassett M.J., Scott J.W., McAuslane H.J. (2002). Mechanisms of resistance in *Lycopersicon* germplasm to the whitefly *Bemisia argentifolii*. Phytoparasitica.

[72] Muñiz M. (2000). Host suitability of two biotypes of *Bemisia tabaci* on some common weeds. Entomol. Exp. Appl..

[73] Muñiz M., Nombela G. (2001). Differential variation in development of B- and Q-biotypes of *Bemisia tabaci* on sweet pepper at constant temperatures. Environ. Entomol..

[74] Iida H., Kitamura T., Honda K. (2009). Comparison of egg-hatching rate, survival rate and development time of the immature stage between B- and Q-biotypes of *Bemisia tabaci* (Gennadius) (Homoptera: Aleyrodidae) on various agricultural crops. Appl. Entomol. Zool..

[75] Tsueda H., Tsuchida K. (2011). Reproductive differences between Q and B whiteflies, *Bemisia tabaci*, on three host plants and negative interactions in mixed cohorts. Entomol. Exp. Appl..

[76] Chu D., Zhang Y.J., Wan F.H. (2010). Cryptic invasion of the exotic *Bemisia tabaci* biotype Q occurred widespread in Shandong Province of China, Fla. Entomologiste (Paris).

[77] Bernays E.A. (1999). When host choice is a problem for a generalist herbivore: experiments with the whitefly *Bemisia tabaci*. Ecol. Entomol..

[78] Bird T.L., Krüger K. (2006). Response of the polyphagous whitefly *Bemisia tabaci* B-biotype (Hemiptera: Aleyrodidae) to crop diversification-influence of multiple sensory stimuli on activity and fecundity. Bull. Entomol. Res..

[79] Mohammad A., Sanjeev G., Agnihotri A.K. (2014). *Ageratum conyzoides* harbours Mungbean yellow mosaic India virus. Plant Pathol. J..

[80] Fiallo‐Olivé E., Tovar R., Navas‐Castillo J. (2016). Deciphering the biology of delta satellites from the New World: maintenance by New World begomoviruses and whitefly transmission. New Phytol..

[81] Zhou X.P., Xie Y., Peng Y., Zhang Z.K. (2003). Malvastrum yellow vein virus, a new *Begomovirus* species associated with satellite DNA molecule. Sci. Bull..

[82] Jiang T., Zhou X.P. (2005). Molecular characterization of a distinct begomovirus species and its associated satellite DNA isolated from *Malvastrum coromandelianum* in China. Virus Gene..

