# Supplementary material for: Impact of leaf trichomes of tomatoes and weeds on the host selection and developmental bioassays of Bemisia tabaci Q and A cryptic species
Source: Heliyon. 2023 Sep 12;9(9):e20077. doi: 10.1016/j.heliyon.2023.e20077 (PMC10559822; doi:10.1016/j.heliyon.2023.e20077)
Supplement: Multimedia component 1 [file mmc1.docx]

**Figure 1S:** Glandular trichomes in section Lycopersicon. Wild accessions have high densities of glandular trichomes that confer resistance to several pests. Panel (**A**) shows the leaflet surface of Solanum habrochaites acc. LA 1777 with high densities of glandular trichome types IV and VI (**B**), and type I (**C**). The surface of Solanum pennellii acc. LA 716 is also covered by type IV trichomes (**D**, **E**) producing and secreting acyl sugars. This accession also has type VI trichomes, but in low density (**F**). Panel (**G**) shows the surface of Solanum lycopersicum cv. Moneymaker. Cultivated tomato has a low density of type VI trichomes (**H**) and type I trichomes. Sometimes, type IV-like trichomes (**I**) are observed on stems, veins, and leaflet edges. White bars represent 500 μm in panels A, C, D, and G. In panels B, E, F, H, and I, bars represent 50 μm and (I) Trichome description according to Luckwill (1943) and revised by Channarayappa et al. (1992).
